# Effects of Resistance Training on the Redox Status of Skeletal Muscle in Older Adults

**DOI:** 10.3390/antiox10030350

**Published:** 2021-02-26

**Authors:** Paulo H. C. Mesquita, Donald A. Lamb, Joshua S. Godwin, Shelby C. Osburn, Bradley A. Ruple, Johnathon H. Moore, Christopher G. Vann, Kevin W. Huggins, Andrew D. Fruge, Kaelin C. Young, Andreas N. Kavazis, Michael D. Roberts

**Affiliations:** 1 School of Kinesiology, Auburn University, Auburn, AL 36849, USA; phc0003@auburn.edu (P.H.C.M.); jsg0061@auburn.edu (J.S.G.); sco0004@auburn.edu (S.C.O.); bar0049@auburn.edu (B.A.R.); jhm0025@auburn.edu (J.H.M.); christopher.vann@duke.edu (C.G.V.); kyoung@auburn.vcom.edu (K.C.Y.); 2 Department of Nutrition, Dietetics and Hospitality Management, Auburn University, Auburn, AL 36849, USA; dal0017@auburn.edu (D.A.L.); huggikw@auburn.edu (K.W.H.); adf0003@auburn.edu (A.D.F.); 3 Department of Cell Biology and Physiology, Edward Via College of Osteopathic Medicine, Auburn, AL 36832, USA

**Keywords:** antioxidants, oxidative damage, oxidative stress, redox homeostasis, exercise

## Abstract

The aim of this study was to investigate the effects of resistance training (RT) on the redox status of skeletal muscle in older adults. Thirteen males aged 64 ± 9 years performed full-body RT 2x/week for 6 weeks. Muscle biopsies were obtained from the vastus lateralis prior to and following RT. The mRNA, protein, and enzymatic activity levels of various endogenous antioxidants were determined. In addition, skeletal muscle 4-hydroxynonenal and protein carbonyls were determined as markers of oxidative damage. Protein levels of heat shock proteins (HSPs) were also quantified. RT increased mRNA levels of all assayed antioxidant genes, albeit protein levels either did not change or decreased. RT increased total antioxidant capacity, catalase, and glutathione reductase activities, and decreased glutathione peroxidase activity. Lipid peroxidation also decreased and HSP60 protein increased following RT. In summary, 6 weeks of RT decreased oxidative damage and increased antioxidant enzyme activities. Our results suggest the older adult responses to RT involve multi-level (transcriptional, post-transcriptional, and post-translational) control of the redox status of skeletal muscle.

## 1. Introduction

Advancing age is characterized by a decline in skeletal muscle mass and function [1] and is associated with the development of several chronic diseases [2]. Different hallmarks of aging have been identified, including genomic instability, loss of proteostasis, and mitochondrial dysfunction [3]. A disruption of the redox homeostasis (i.e., the balance between the production and removal of reactive species) can lead to a state of oxidative stress, which has been considered one of the cellular mechanisms driving the aging process [4,5,6,7].

Several studies have reported increased oxidative stress with aging [8,9,10]. Although reactive species are now recognized as important regulators of several signaling pathways [11], their overproduction can cause damage to different cellular components. Essential in preventing such damage and maintaining the redox homeostasis in skeletal muscle are the antioxidant enzymes superoxide dismutase 1 (SOD1) and 2 (SOD2), catalase (CAT), glutathione peroxidase (GPX), and glutathione reductase (GSR) [12]. Together, these endogenous antioxidants constitute a defense and repair system that works against the potentially harmful effects of reactive species. In addition, a family of stress-induced proteins called heat shock proteins (HSPs) have been shown to be responsive to oxidative stress and to have a role in the skeletal muscle defense system [13,14,15].

Exercise has been considered a therapeutic tool to reduce chronic oxidative stress. Indeed, a review conducted by de Sousa et al. [16] suggested that exercise training promotes increased antioxidant capacity and decreased oxidative damage, regardless of the type of exercise, volume, and intensity. Nevertheless, studies investigating the effects of resistance training (RT) on antioxidant and oxidative stress parameters are scarce. A recent systematic review and meta-analysis conducted by Cuyul-Vásquez et al. [17] sought to elucidate the effects of RT on redox homeostasis in older individuals. While their results suggested that RT has no beneficial effects on various biomarkers, the authors emphasize that the results should be interpreted with caution because of the limited number and low methodological quality of studies available. In addition, Cuyul-Vásquez et al. [17] highlight the fact that most studies interrogated plasma, which may not reflect the redox signaling in skeletal muscle [18] and may not be appropriate for determining antioxidant enzymatic activity [19].

To the best of our knowledge, no study to date has performed a thorough investigation on the effects of RT on antioxidants and oxidative damage in skeletal muscle of older humans using a transcriptional, post-transcriptional, and post-translational approach. Therefore, the purpose of this study was to investigate the effects of RT on the redox status of skeletal muscle of older adults. Specifically, we examined the effects of 6 weeks of RT on the mRNA, protein, and enzymatic activity of several endogenous antioxidants and markers indicative of oxidative stress. Moreover, we measured protein levels of certain HSPs given their association with oxidative stress. For referencing purposes, we also compared the assayed parameters to a group of college-aged individuals in order to examine if RT was capable of restoring the assayed biomarkers to “youth-like” levels. Our study demonstrated that 6 weeks of RT significantly decreased lipid peroxidation and increased antioxidant enzymatic activities. Further, RT increased the mRNA levels of all assayed antioxidant genes, and either did not change or decreased the protein levels of antioxidants.

## 2. Materials and Methods

### 2.1. Ethical Approval

This study is a secondary analysis of two previous studies approved by the Institutional Review Board at Auburn University. The first study (Protocol # 19-249 MR 1907) investigated the effects of peanut protein supplementation and RT on the muscle mass and strength of older participants. The second study (Protocol # 19-245 MR 1907) aimed to explore the effects of the high-load low-volume and high-volume low-load RT paradigms on muscle hypertrophy in college-aged males. All participants provided written consent prior to participation and both studies conformed to standards set by the Declaration of Helsinki. A two-way repeated measures analysis of variance (ANOVA) was conducted to analyze the effects of peanut protein supplementation on the variables investigated in the current study. The results showed no effects of supplementation (*p* > 0.050) for most variables, and, therefore, the supplemented and non-supplemented groups were combined for analysis. The variables that were affected by supplementation (*p* < 0.050; total antioxidant capacity (TAC) and GSR) are highlighted and discussed in the results and discussion sections.

### 2.2. Participants

Thirteen older males (age = 64 ± 9 years) without recent RT experience were recruited to participate in this study. Inclusion criteria required participants to be 50–80 years old, have not been participating in structured RT for at least three months prior, and to abstain from nutritional supplementation for one month prior to enrollment. Participants also had to be free of overt cardio-metabolic diseases (e.g., type II diabetes, severe hypertension, heart failure) or conditions that precluded the collection of a skeletal muscle biopsy. Data from ten recreationally resistance-trained college-aged males (age = 23 ± 3 years, average training experience = 7 years) were also obtained and are presented in the graphs and Table 1 and Table 2 below.

### 2.3. Study Design

Participants visited the laboratory on two separate occasions prior to beginning the RT program. The first visit consisted of a battery of assessments including height, body mass, full-body dual-energy x-ray absorptiometry (DXA), ultrasound of the right leg vastus lateralis (VL), and a right leg strength assessment using an isokinetic dynamometer. On the second visit, a biopsy of the VL was taken from the right leg for posterior molecular analyses. Participants then completed the 6-week RT program and reported back to the laboratory for post-training assessments 72 h following their last training bout.

### 2.4. Testing Sessions

A complete description of the testing procedures can be found in Lamb et al. [20]. Briefly, participants reported to the laboratory following an overnight fast, body mass and height were assessed, and images were captured from the right leg VL using real-time B-mode ultrasonography (LOGIQ S7 Expert, GE Healthcare, USA). Thereafter, participants underwent a full-body DXA scan (Lunar Prodigy; GE Corporation, Fairfield, CT, USA) for the determination of fat-free mass (FFM) and fat mass (FM). Following the DXA scan, participants performed maximal isokinetic right leg extensions at 60°/s and 120°/s.

### 2.5. Muscle Biopsies

Muscle biopsies were taken from the right leg VL before the RT program (Old-Pre) and 72 h following the last training session (Old-Post) for the older participants, and in the basal state for the college-aged group (Young). A 5-gauge needle was used to obtain biopsies of the right leg VL as previously described by our laboratory [21]. Following biopsies, tissue was rapidly teased of blood and connective tissue, flash-frozen using liquid nitrogen, and subsequently stored at −80 °C for future use.

### 2.6. Resistance Training (RT) Program

The RT program consisted of a whole-body workout performed twice weekly for six weeks. Each session was composed of the following exercises: (1) leg press, (2) leg extensions, (3) leg curls, (4) barbell bench press, (5) cable pulldowns. Participants performed 3 sets of 10–12 repetitions for each exercise with 1 min of rest between sets. Participants were asked to rate the level of difficulty for each set (0 = easy, 10 = hard), and weight was adjusted accordingly to ensure a 7–9 rating. All exercises and training sessions were recorded, and all participants increased the weight lifted throughout the program. More in-depth details about training volume and progression can be found in Lamb et al. [20].

### 2.7. mRNA Analysis

A subset of ten older and eight younger participants was used for mRNA analysis. Approximately 20 mg of muscle was placed in 500 μl of Ribozol (Ameresco, Solon, OH, USA) and RNA isolation proceeded following the manufacturer’s instructions. RNA concentrations were determined in duplicate using a NanoDrop Lite (Thermo Fisher Scientific, Walthan, MA, USA), and cDNA (2 μg) was synthesized using a commercial qScript cDNA SuperMix (Quanta Biosciences, Gaithersburg, MD, USA). RT-qPCR was performed in an RT-PCR thermal cycler (Bio-Rad) using SYBR green-based methods with gene-specific primers designed with primer designer software (Primer3Plus, Cambridge, MA, USA). For all primer sets, pilot qPCR reactions and melt data indicated that only one amplicon was present. The forward and reverse primer sequences of all genes are listed in Table 1. Fold change values were performed using the 2^ΔΔCq^ method, where 2^ΔCq^ = 2 ^ (housekeeping gene (HKG) Cq—gene of interest Cq) and 2^ΔΔCq^ (or fold change) = (2^ΔCq^ value/2^ΔCq^ average of Old-Pre). Glyceraldehyde-3-phosphate dehydrogenase (GAPDH) was used as a reference gene.

### 2.8. Western Blotting

Approximately 20 mg of muscle tissue was homogenized in lysis buffer (25 mM Tris, pH 7.2, 0.5% Triton X-100, 1x protease inhibitors) using tight-fitting plastic pestles. Samples were then centrifuged at 500× *g* for 5 min at 4 °C. Supernatants were placed in new cryotubes, and protein concentrations were determined from supernatants using a commercially available BCA kit (Thermo Fisher Scientific, Walthan, MA, USA). Supernatants were then prepared for Western blotting using 4x Laemmli buffer and distilled water (diH2O).

Twelve microliters (12 μL) of Western blot preps were loaded on gradient SDS-polyacrylamide gels (4–15% Criterion TGX Stain-free gels; Bio-Rad Laboratories; Hercules, CA, USA), and proteins were separated by electrophoresis for approximately 45 min at 200 V. Subsequently, proteins were transferred to pre-activated PVDF membranes (Bio-Rad Laboratories) for 2 h at 200 mA. Membranes were then Ponceau stained, quickly washed with diH_2_O, dried, and digitally imaged with a gel documentation system (UVP, LLC, Upland, CA, USA). Following Ponceau imaging, methanol was used to re-activate membranes, which were then blocked with nonfat milk (5% *w*/*v* diluted in Tri-buffered saline with 0.1% Tween 20 (TBST)) for 1 h, washed three times in TBST (5 min per wash), and incubated for 1 h with primary antibodies (1:2000 *v*/*v* dilution in TBST with 5% BSA). Primary antibodies included: SOD1 (GeneTex Cat# GTX100554, RRID:AB_10618670), SOD2 (GeneTex Cat# GTX116093, RRID:AB_10624558), CAT (GeneTex Cat# GTX110704, RRID:AB_1949848), GPx-1 (GeneTex Cat# GTX116040, RRID:AB_2037097), HSP60 (Abcam Cat# ab46798, RRID:AB_881444), HSP70 (Abcam Cat# ab79852, RRID:AB_1603786), HSP90 (Abcam Cat# ab13495, RRID:AB_1269122), and 4-hydroxynonenal (4HNE) (Abcam Cat# ab46545, RRID:AB_722490). Protein carbonyl levels were assessed using the Oxyblot protein oxidation detection kit (Millipore, Billerica, MA; #S7150). Validation of the antibodies was previously reported [22,23,24,25,26]. Afterwards, membranes were washed in TBST and incubated with anti-rabbit secondary antibody (Cell Signaling Technology Cat# 7074, RRID:AB_2099233) for 1 h. Membranes were washed in TBST again, developed using chemiluminescent substrate (Millipore; Burlington, MA, USA), and digitally imaged in a gel documentation system (UVP, LLC, Upland, CA, USA). ImageJ software (NIH, Bethesda, MD, USA) was used to obtain the raw density of target bands. The most prominent band (~45 kD) in the Ponceau stains was used to normalize the target bands. The values were then divided by the mean of the Old-Pre group to obtain fold-change values. 4HNE blots were analyzed from approximately 100 to 17 kD, while the whole lane (approximately 245 to 20 kD) was analyzed for protein carbonyl blots.

### 2.9. Enzymatic Activities

Muscle lysates were used to determine total antioxidant capacity (TAC) (Cayman, MI, USA, Cat# 709001), and the enzymatic activities of catalase (Cayman, MI, USA, Cat# 707002), glutathione peroxidase (Cayman, MI, USA, Cat# 703102), and glutathione reductase (Cayman, MI, USA, Cat# 703202) according to the manufacturer’s instructions for each assay kit. Enzymatic activity was normalized by the quantity of protein (mg) used in each essay. Superoxide dismutase activity was also assessed using a colorimetric assay kit (Cayman, MI, USA, Cat# 706002), but the interference of the reagents used in the homogenization of muscle samples prevented the acquisition of reliable data. Therefore, SOD activity is not reported herein.

### 2.10. Statistics

Data are expressed as mean ± standard deviation (SD) values, and 95% confidence intervals are presented for differences between time-points and between groups. Shapiro–Wilk tests were used to assess the distribution of data for each dependent variable. Dependent sample *t*-tests were used to analyze pre x post data for the older group. In addition, independent sample *t*-tests were used to compare the Old-Pre and Old-Post groups to the Young group. Variables that did not present a normal distribution were analyzed using Wilcoxon signed rank or Mann–Whitney U tests for dependent and independent variables, respectively. Statistical significance was established at *p* < 0.050. All statistical analyses were performed using SPSS v21.0 (IBM Corp, Armonk, NY, USA).

## 3. Results

### 3.1. Participant Characteristics and Training Adaptations

Participant characteristics and training adaptations can be found in Table 2. Notably, the RT program increased the following variables in older participants: FFM (*p* < 0.001, 95% CI (0.27, 1.24)), VL thickness (*p* = 0.040, 95% CI (0.01, 0.25)), and knee extension torque at 60°/s (*p* = 0.033, 95% CI (1.77, 36.28)) and 120°/s (*p* = 0.022, 95% CI (2.89, 31.00)). In addition, there was a decreasing trend for FM (*p* = 0.081, 95% CI (−1.02, 0.07)).

### 3.2. mRNA Expression

**Figure 1:**
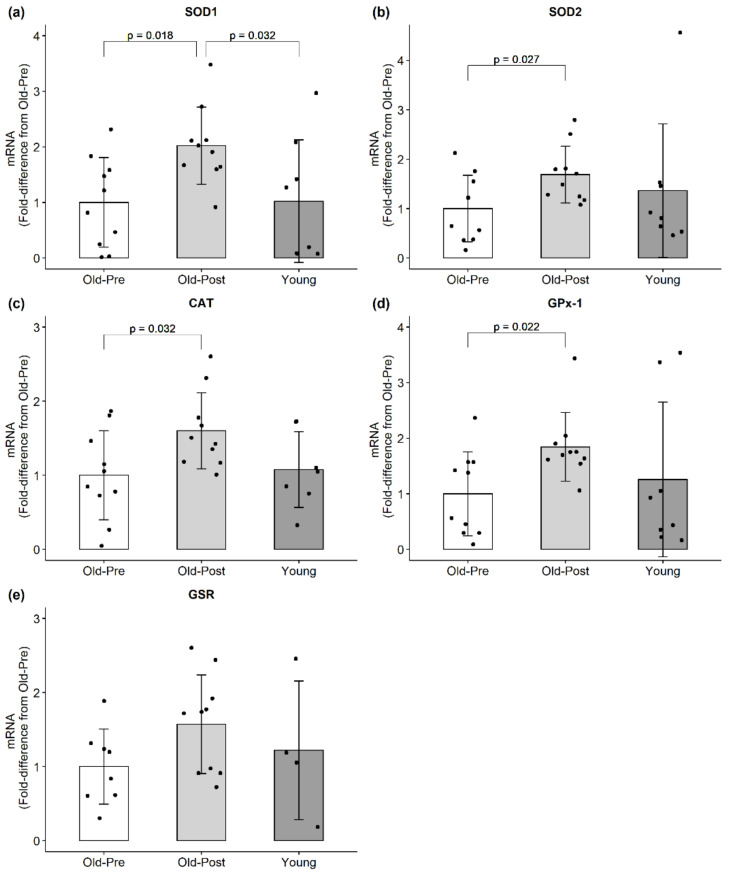
mRNA expression of endogenous antioxidants. All data are presented as mean ± SD values, and individual data points representing each participant is superimposed on each bar graph. (**a**) SOD1; (**b**) SOD2; (**c**) CAT; (**d**) GPx-1; (**e**) GSR.

Muscle mRNA expression is presented in Figure 1. Resistance training significantly increased the mRNA expression of all assayed antioxidants (SOD1: *p* = 0.018, 95% CI (0.22, 1.82); SOD2: *p* = 0.027, 95% CI (0.10, 1.28); CAT: *p* = 0.032, 95% CI (0.07, 1.13); GPx-1: *p* = 0.022, 95% CI (0.10, 1.59)) except for GSR, which presented a trend to increase but did not reach statistical significance (*p* = 0.092, 95% CI (−0.11, 1.12)). Notably, the reference gene (GAPDH) was not affected from Pre to Post in the older participants (*p* = 0.523).

### 3.3. Protein Levels

**Figure 2:**
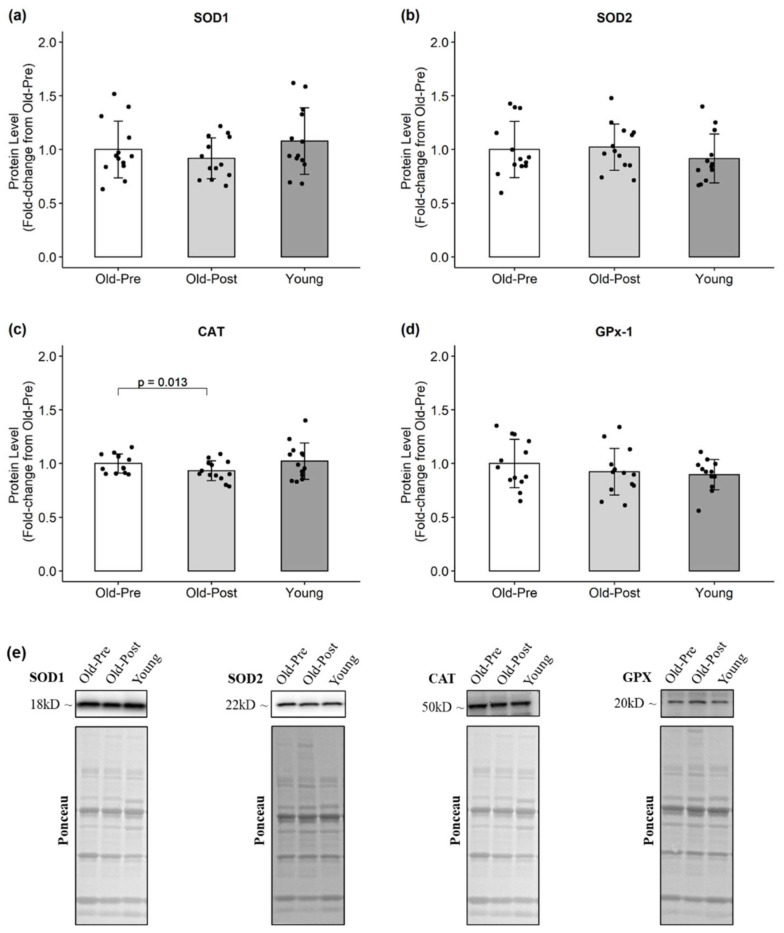
Protein levels of endogenous antioxidants. All data are presented as mean ± SD values, and individual data points representing each participant are superimposed on each bar graph. (**a**) SOD1; (**b**) SOD2; (**c**) CAT; (**d**) GPx-1; (**e**) representative Western blots.

**Figure 3:**
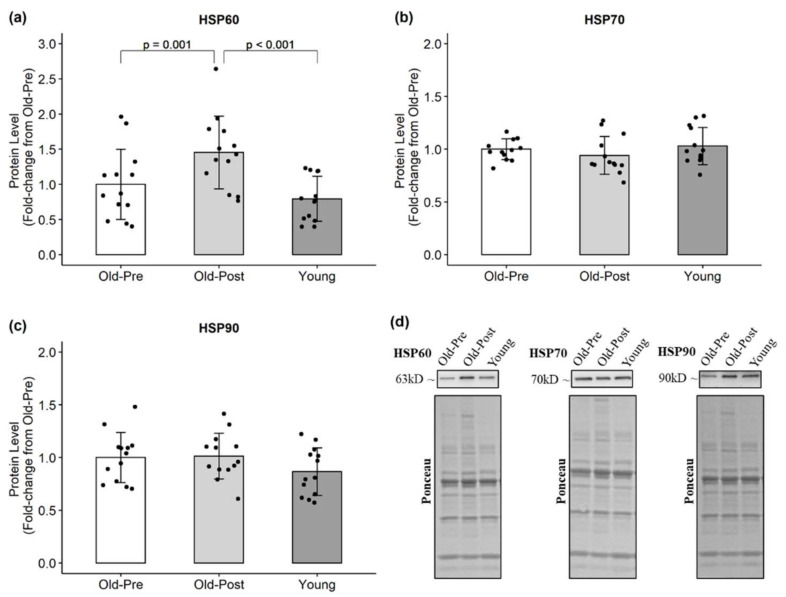
Protein levels of HSPs. All data are presented as mean ± SD values, and individual data points representing each participant are superimposed on each bar graph. (**a**) HSP60; (**b**) HSP70; (**c**) HSP90; (**d**) representative Western blots.

**Figure 4:**
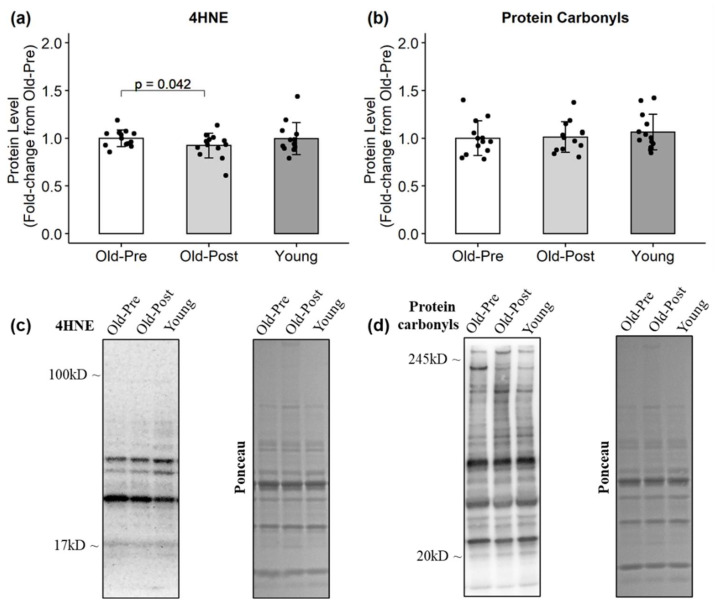
Protein levels of oxidative damage markers. All data are presented as mean ± SD values, and individual data points representing each participant are superimposed on each bar graph. (**a**) 4HNE; (**b**) protein carbonyls; (**c**,**d**) representative Western blots.

In the older group, resistance training significantly decreased CAT (*p* = 0.013, 95% CI (−0.02, −0.12)) (Figure 2) and increased HSP60 protein levels (*p* = 0.001, 95% CI (0.22, 0.68)) (Figure 3). Regarding markers of oxidative damage, 4HNE decreased following RT (*p* = 0.042, 95% CI (0.01, 0.15)), but protein carbonyl levels remained unchanged (*p* = 0.852) (Figure 4).

### 3.4. Enzymatic Activity

**Figure 5:**
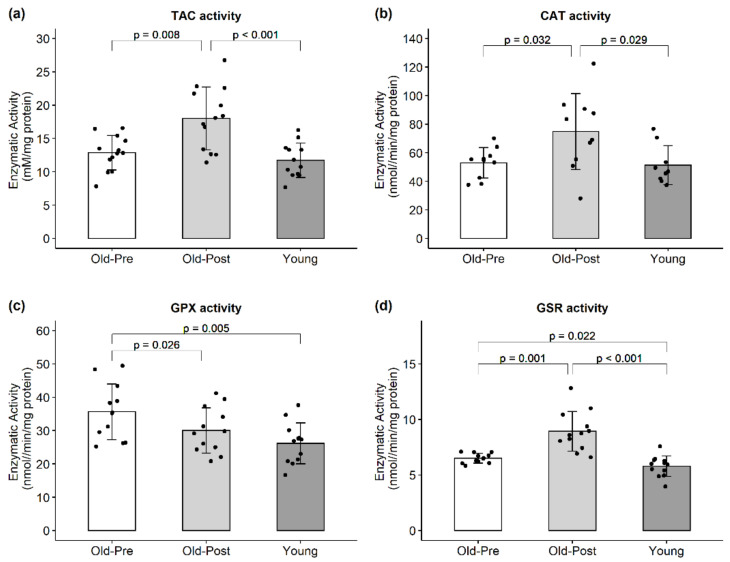
Enzymatic activity of endogenous antioxidants. All data are presented as mean ± SD values, and individual data points representing each participant are superimposed on each bar graph. (**a**) Total antioxidant capacity; (**b**) CAT activity; (**c**) GPX activity; (**d**) GSR activity.

RT increased the enzymatic activity of CAT (*p* = 0.032, 95% CI (2.28, 41.56)). On the other hand, RT decreased GPX activity (*p* = 0.026, 95% CI (−0.80, −10.35)) (Figure 5). TAC and GSR activities exhibited an interaction effect in response to the peanut protein supplementation and RT (*p* = 0.037 and *p* = 0.005, respectively) where the peanut protein supplemented group had a lower increase in TAC and GSR activities than the placebo. However, when data were analyzed collectively, RT increased TAC (*p* = 0.008, 95% CI (1.60, 8.73)) and GSR (*p* = 0.001, 95% CI (1.24, 3.61)) (Figure 5).

## 4. Discussion

The aim of this study was to investigate the effects of RT on the redox status of skeletal muscle in older adults. Previous studies by our group in a separate cohort of older individuals showed that 10 weeks of RT led to an improvement in several markers of muscle metabolism [27] and mitochondrial remodeling [28]. In line with these findings, 6 weeks of RT promoted positive adaptations in strength, body composition, and in the redox status of skeletal muscle. Specifically, our results show that RT increased mRNA expression, decreased (e.g., CAT) or did not alter protein content, and increased the enzymatic activity of the antioxidants investigated. In addition, RT increased the protein levels of HSP60 and decreased lipid peroxidation.

Our results align with prior literature that indicates that the mRNA levels of antioxidants seem to be mainly unaltered in skeletal muscle with aging [29,30,31,32]. However, data regarding the effects of exercise training on the mRNA levels of antioxidants are limited and equivocal, especially in relation to RT adaptations. Ryan et al. [10,33], for example, found unaltered mRNA expression of antioxidants in the skeletal muscle of young and aged rats exposed to chronic repetitive loading. The significant increase in mRNA expression observed in the present study is in agreement with a previous study conducted by García-López et al. [34]. The authors found increased mRNA expression of SOD1, SOD2, CAT, and GPx in peripheral blood mononuclear cells of middle-aged men after 21 weeks of RT. Therefore, RT seems to promote cellular stress of sufficient magnitude to activate signaling pathways and increase antioxidant transcript levels in older adults, and this may occur over multiple tissues.

Although we observed significant increases in mRNA levels of the antioxidants analyzed, protein levels were either unaltered or decreased following RT. Although surprising at first, several authors have reported mRNA levels to be a poor indicator of protein content or enzymatic activity of antioxidants [19,34,35]. García-Lopez et al. [34], for example, found increased mRNA but unaltered protein levels for most of the antioxidants investigated. The authors hypothesized that the discrepancies observed could be related, at least in part, to differences in mRNA stability or translational efficiency. Most studies available to date have found no change in antioxidant protein levels in older subjects in response to RT. For instance, Parise et al. [36] found no change in SOD1, SOD2, and CAT protein content in the skeletal muscle of older participants after 14 weeks of RT. The protein levels of SOD1, SOD2, CAT, and GPx-1 were also unaltered in the skeletal muscle of rats exposed to chronic repetitive loading [10,33]. There are also reports of decreased SOD1 [37] and a trend for CAT protein levels to decrease [38] in response to RT. Although it is difficult to reconcile the reasons for the decrease in protein content of antioxidants, especially with increased mRNA levels, we speculate that this may be related to increased antioxidant enzymatic activity, which may increase antioxidant efficiency, coupled with the decreased lipid peroxidation observed in the present study. Specifically, the increased antioxidant efficiency could have decreased oxidative stress, which in turn reduces the need for high protein levels of antioxidants. Another explanation is that an initial decrease in protein content may have initiated a negative feedback signal, increasing both gene expression and enzymatic activities of various antioxidants. While it remains uncertain as to why these paradoxical observations were made, these findings clearly demonstrate that a complex interrelationship exists between antioxidant mRNA, protein, and enzyme activity levels, which should be investigated further.

It is well-documented that aging is associated with an increase in oxidative damage to lipids, proteins, and DNA [9,10,15,39]. However, our results did not show any significant differences in lipid peroxidation or protein carbonyl levels between older and younger participants. This discrepancy could be related to the age of the participants included in the present study. Although our subjects were of advanced age (mean = 64 years), previous studies have shown that oxidative damage was significantly higher only in subjects considerably older (>66–70 years) [9,39]. Even though the older group did not display greater lipid peroxidation at baseline compared to the younger group, 6 weeks of RT significantly decreased 4HNE levels. Parise and colleagues have previously shown that RT is capable of decreasing oxidative damage to DNA [36] but not to proteins [40] in older participants. Resistance training has also been shown to decrease lipid peroxidation levels in the plasma of young participants [41] and in the skeletal muscle of aged rats [10,33].

Despite reports that aged skeletal muscle fails to upregulate HSPs following acute exercise, possibly due to heightened basal expression, we found increased HSP60 protein levels after 6 weeks of RT. HSPs help protect against the potentially harmful effects of ROS production [13,42] and have been shown to increase in response to RT [37,43,44]. Therefore, the increase in HSP60 could have been an adaptation to transient increases in ROS production, helping to protect the skeletal muscle cells from subsequent bouts of oxidative stress. In addition, it has been suggested before that high levels of oxidative stress may impair the exercise-induced increase in HSPs [37]. Therefore, the increased HSP levels observed in the present study could also be a consequence of diminished oxidative stress.

The enzymatic activity of antioxidants in skeletal muscle increases with aging [8,31], supposedly as an adaptation to increased chronic oxidative stress. In the present study, GPX and GSR activities were higher in the Old-Pre versus Young participants, despite no significant difference in markers of oxidative damage between these two groups. Our results show that 6 weeks of RT increased total antioxidant capacity in the skeletal muscle of older subjects, as well as CAT and GSR activities. Our data agree with a previous study conducted by Parise et al. [40], which also showed increased antioxidant enzyme activity in older males after RT. Because acute RT has been shown to promote a transient increase in oxidative stress [45,46,47,48], the increase in antioxidant activities observed could be an adaptation to repeated exposure to elevated oxidative stress. Surprisingly, GPX activity was decreased following RT. Ryan et al. [10] also observed the same response in the skeletal muscle of rats after chronic loading and speculated that either GPX activity was suppressed by the high levels of H_2_O_2_ or that the increased CAT activity was sufficient to counteract the increased H_2_O_2_ production.

## 5. Conclusions

In conclusion, we showed that 6 weeks of RT promoted beneficial adaptations in the redox status of the skeletal muscle in older adults. RT significantly decreased lipid peroxidation and increased antioxidant enzymatic activities. Therefore, RT may be a viable approach to counteract a possible age-related disruption of skeletal muscle redox homeostasis in older adults. Furthermore, our findings suggest a multilevel control of the antioxidant system response to RT, involving transcriptional, post-transcriptional, and post-translational controls. Thus, researchers should exercise caution when investigating and interpreting results from only one of the levels of control. It should be noted that the duration of the RT program adopted in the present study was very short, and that long-term redox adaptations might differ from those observed herein. Moreover, future studies should investigate the effects of different RT protocols, such as low-load high-volume training, which have been suggested to promote greater metabolic adaptations [49,50].

## Figures and Tables

**Table 1:** PCR primer sequences for mRNA analysis of antioxidant genes.

Gene	Primer Sequences	Amplicon Length	Position	NCBI Ref. Seq.
SOD1	**FP (5′ → 3′):** TGTGGCCGATGTGTCTATTGAA **RP (5′ → 3′):** CACCTTTGCCCAAGTCATCTG	109 bp	**FP:** 430–451 **RP:** 519–539	NM_000454
SOD2	**FP (5′ → 3′):** GTTGGGGTTGGCTTGGTTTC **RP (5′ → 3′):** GCCTGTTGTTCCTTGCAGTG	89 bp	**FP:** 511–530 **RP:** 580–599	NM_000636
CAT	**FP (5′ → 3′):** CTGACTACGGGAGCCACATC **RP (5′ → 3′):** AGATCCGGACTGCACAAAGG	92 bp	**FP:** 1540–1559 **RP:** 1612–1631	NM_001752
GPx-1	**FP (5′ → 3′):** ACGAGGGAGGAACACCTGAT **RP (5′ → 3′):** TCTGGCAGAGACTGGGATCA	80 bp	**FP:** 775–794 **RP:** 835–854	NM_000581
GSR	**FP (5′ → 3′):** AAAAAGTACACCGCCCCACA **RP (5′ → 3′):** ATCTGGCTCTCATGAGGGGT	71 bp	**FP:** 576–595 **RP:** 1612–1631	NM_000637
GAPDH	**FP (5′ → 3′):** AACCTGCCAAATATGATGAC **RP (5′ → 3′):** TCATACCAGGAAATGAGCTT	193 bp	**FP:** 828–847 **RP:** 1001–1020	NM_002046

FP, forward primer; RP, reverse primer; SOD1, superoxide dismutase 1; SOD2, superoxide dismutase 2; CAT, catalase; GPx-1, glutathione peroxidase 1; GSR, glutathione reductase; GAPDH, glyceraldehyde 3-phosphate dehydrogenase.

**Table 2:** Participant characteristics and training adaptations.

	Old-Pre	Old-Post	Young
Body mass (kg)	89.8 ± 11.7	90.1 ± 11.5	89.7 ± 11.7
FFM (kg)	59.2 ± 6.1	59.9 ± 6.4 *	69.3 ± 8.0 *^#^
FM (kg)	27.4 ± 7.5	27.0 ± 7.2	17.2 ± 7.2 *^#^
VL thickness (cm)	2.18 ± 0.4	2.31 ± 0.3 *	2.8 ± 0.3 *^#^
mCSA (cm^2^)	148.9 ± 21.4	151.1 ± 21.7	201.3 ± 28.9 *^#^
Knee ext—60°/s (N.m)	154 ± 55	173 ± 41 *	229 ± 46 *^#^
Knee ext—120°/s (N.m)	125 ± 41	142 ± 34 *	191 ± 36 *^#^

Data are presented as mean ± standard deviation; FFM, fat-free mass; FM, fat mass; VL, vastus lateralis, mCSA, muscle cross-sectional area; ext, extension. *, different from Old-Pre (*p* < 0.050); ^#^, different from Old-Post (*p* < 0.050).

## Data Availability

The datasets used and/or analyzed during the current study are available from the corresponding authors.
